# Product recovery in microbial biotechnology An annotated selection of World Wide Web sites relevant to the topics in microbial biotechnology

**DOI:** 10.1111/1751-7915.12122

**Published:** 2014-04-08

**Authors:** Lawrence P Wackett

**Affiliations:** Department of Biochemistry, Molecular Biology and Biophysics, BioTechnology Institute, University of MinnesotaSt. Paul, MN, 55108, USA

## Biochemical product recovery

### http://www.rpi.edu/dept/chem-eng/Biotech-Environ/DOWNSTREAM/fixed.htm

This site provides a good overview of the different aspects of product recovery in biotechnology, from proteins to small molecules.

## Downstream processing

### http://en.wikipedia.org/wiki/Downstream_processing

Downstream processing refers to the recovery and purification of products from biotechnological processes. This short article provides links to further information on different aspects of the process.

## *In situ* product recovery

### https://www.cell.com/trends/biotechnology/abstract/0167-7799(84)90022-2

This review article discusses innovative methods for bioprocess product recovery.

## Purification of isoprene from renewable resources

### https://www.google.com/patents/WO2011075534A2?cl=en

Isoprene boils at the growth temeprature of *Escherichia coli* cells, and this makes for a convenient recovery system in which isoprene gas can be recovered from fermentors.

## Membrane extraction system for ethanol

### http://www1.eere.energy.gov/manufacturing/intensiveprocesses/pdfs/adv_water_removal_mse.pdf

There is a significant cost in money and energy to extract ethanol biofuels from aqueous media as ethanol is highly soluble in water and has a fairly high boiling point. In this context, this file describes a membrane technology for removing ethanol from fermentation broths.

## Ethanol stripping from fermentors

### https://workspace.imperial.ac.uk/centreforprocesssystemsengineering/Public/MSc%20Thesis/2010%20MSc%20Thesis/2010.2%20-%20Andrew%20Amenaghawon%20.pdf

Because ethanol recovery is a major cost in money and energy, less expensive methods have been sought. This thesis deals with using gas stripping methods.

## A survey of separation systems for ethanol

### http://scholar.lib.vt.edu/theses/available/etd-03052009-175635/unrestricted/Thesis_ETD8.pdf

This thesis describes a flash evaporation process for efficient removal of ethanol from aqueous fermentation broths.

## Acetone-butanol-ethanol fermentation recovery process

### http://pubs.acs.org/doi/abs/10.1021/ef401706k

This paper describes a novel membrane system for solvent recovery from the *Clostridium acetobutylicum*-mixed solvent fermentation.

## Method of recovering 1,3-propanediol from fermentation broth

### http://www.google.com/patents/US6479716

1,3-Propanediol is an important commodity chemical made in large-scale fermentation processes. The product was initially recovered by solvent extraction. This patent describes the use of a cationic resin to bind the chemical and remove it from the fermentation broth.

## Extraction of antibiotics

### http://www.westfalia-separator.com/applications/chemical-pharmaceutical-technology/extraction/antibiotics.html

This commercial website describes a process for extracting products from broths with a high solid content, such as those found with fungal mycelia.

## Vancomycin extraction

### http://www.dynamicadsorbents.com/vancomycin.htm

This commercial website describes an alternative to standard ion exchange resins, using alumina in which the pore sizes can be adjusted to enhance recovery of products per unit material.

## Supercritical fluids extraction in bioprocesses

### http://www.jbiochemtech.com/index.php/jbt/article/viewFile/JBT215/51

This review article discusses the uses of supercritical carbon dioxide in product recovery for biotechnology.

## Recovery of vanillin from fermentation media

### http://blogs.rsc.org/gc/2011/08/01/sustainable-recovery-of-pure-natural-vanillin-from-fermentation-media-in-one-step/

This green chemistry blog briefly describes the use of a membrane evaporation system to quickly remove vanillin from fermenation broths without the use of solvents.

